# Diverse Approaches to Creating and Using Causal Loop Diagrams in Public Health Research: Recommendations From a Scoping Review

**DOI:** 10.3389/phrs.2021.1604352

**Published:** 2021-12-14

**Authors:** Lori Baugh Littlejohns, Carly Hill, Cory Neudorf

**Affiliations:** Community Health and Epidemiology, University of Saskatchewan, Saskatoon, SK, Canada

**Keywords:** scoping review, causal loop diagrams, public health research, methods, complex systems thinking

## Abstract

**Objectives:** Complex systems thinking methods are increasingly called for and used as analytical lenses in public health research. The use of qualitative system mapping and in particular, causal loop diagrams (CLDs) is described as one promising method or tool. To our knowledge there are no published literature reviews that synthesize public health research regarding how CLDs are created and used.

**Methods:** We conducted a scoping review to address this gap in the public health literature. Inclusion criteria included: 1) focused on public health research, 2) peer reviewed journal article, 3) described and/or created a CLD, and 4) published in English from January 2018 to March 2021. Twenty-three articles were selected from the search strategy.

**Results:** CLDs were described as a new tool and were based upon primary and secondary data, researcher driven and group processes, and numerous data analysis methods and frameworks. Intended uses of CLDs ranged from illustrating complexity to informing policy and practice.

**Conclusion:** From our learnings we propose nine recommendations for building knowledge and skill in creating and using CLDs for future public health research.

## Introduction

There is a trend in public health research for the application of complex systems thinking methods and tools [1–3]. We conceptualize public health research from this perspective in terms of examining systems that are complex webs of sectors, institutions, people, structures, and interventions that aspire to maintain and improve population health. Furthermore, we value public health research that is “based on the principles of social justice, attention to human rights and equity, evidence-informed policy and practice, and addressing the underlying determinants of health” [4].

There are published review articles regarding complex systems thinking methods used in public health research and together these paint a broad landscape [2, 3, 5–10]. In this literature, there is clear support for using qualitative system mapping and in particular, causal loop diagrams (CLDs) as analytical tools to embed complex systems thinking. The origins of the use of CLDs emanate from the system dynamics branch of systems science founded by Forrester [11] and CLDs are needed because “we live in a complex of nested feedback loops” [12]. One example of using a CLD in public health research is a study of factors that influenced health promotion policy and practice in a regional public health system [13]. Here, the CLD was useful because “feedback mechanisms can be seen as leverage points to strengthen systems” and to “identify potential opportunities to disrupt or slow down vicious feedback mechanisms or amplify those that are virtuous cycles.” At the time of this study (2018), there were few examples of CLDs in public health literature [14–21].

To our knowledge there are no published reviews that synthesize public health research in terms of how CLDs are created and used. We were motivated to conduct a literature review to determine how CLD methodology could be used to identify leverage points in local public health systems to strengthen the response to COVID-19 in Canada. The aim of this paper is to address this gap in the literature and synthesize knowledge from recent innovations for our research and contribute to knowledge development. We posed two research questions: 1) How are CLDs created and used in recent (>2018) public health research? 2) What recommendations emerge regarding how to create and use CLDs in public health research?

## Methods

A scoping review was chosen for this study in order “to examine how research is conducted” and “to provide an overview or map of the evidence” [22]. A narrative synthesis approach was utilized as the topic required exploration more than explanation and human and time resources were limited [23]. Key issues identified by Byrne [24] to strengthen the review were addressed such as ensuring transparency in search strategy and data extraction, analysis and synthesis.

### Search Strategy

Literature was searched using the Scopus and PubMed databases and used the following search terms: causal loop diagram*, complex*, system* thinking, method*, tool, approach, research, and public health. Inclusion criteria were 1) public health research, 2) peer reviewed journal article, 3) described or created a CLD as a research method, and 4) published in English from January 2018 to March 2021. The key objective was to find state-of-the-field examples of CLDs, therefore, extensive hand searches of references was completed. It is important to note that piloting this search strategy uncovered numerous articles that only mentioned CLDs and did not explicitly meet the criteria of “described or created a CLD as a research method.” While we set out to use PRISMA guidelines we deemed it unnecessary given the search strategy quickly became one of including *all* articles that meet our inclusion criteria.

### Data Extraction and Analysis

Study selection was conducted by one author (LBL) while appraisal and duplicate independent data extraction and validation was conducted by two authors (LBL and CH). CN provided input throughout the study and facilitated discussion about any differences. Data extraction followed these six categories:

1) Research aim,
2) Description of complex systems thinking,
3) Why a CLD was selected as a method,
4) How the CLD was created,
5) How the CLD was used, and
6) Recommendations for future research using CLDs.

Two authors (LBL and CH) extracted verbatim text that aligned with the extraction categories and these were saved to a spreadsheet. Both authors reviewed the spreadsheet in its entirety, discussed individual articles to gain clarity, and wrote summary paragraphs to identify high level themes. Following this, for each article, summary statements were written for the six extraction categories and a table was created. The two authors reviewed each other’s summaries for accuracy and revisions were made. Finally, directed content analysis was used to interpret extracted data “through systematic classification of coding and identifying themes and patterns” [25].

## Results

We found 23 articles in total that met our inclusion criteria. A list of these articles and summary statements are provided in Table 1. This section answers our first research question: How are CLDs created and used in recent (>2018) public health research? The organization of this section mirrors the six data extraction categories indicated above.

**TABLE 1:** Summary statements of extracted data (Canada, 2021).

First author/citation	Research aim	Description: complex systems thinking	Why a CLD was selected as a method	How the CLD was created	How the CLD was used
Allender [47]	To report on insight gathered during development, implementation, and evaluation of the first 2 years in a systems-based childhood obesity prevention initiative that was inspired by community based system dynamics	Complexity hampers traditional approaches to improving population health; need to conceptualize health as the result of actors and “interdependent elements connected at multiple levels”; initiatives such as obesity prevention need to address feedback loops that can lead to policy resistance, time delays that influence long term system change, and accumulations and their rates of change	To create a visual model of the causes and effects of childhood obesity	Local behavioral data (collected using a monitoring system and electronic tablets) was used to support the creation of CLD during group model building in communities	Create engagement (“whole community”) and momentum for the intervention communities to take action on childhood obesity
Araz [45]	To analyze potential “real world” impacts of policy interventions on improving roadway safety regarding drugged driving behavior, road environment, and policy through system dynamics modeling	Driver behavior was described as a complex system given the dynamic interrelationships and multidimensional variables associated with driving behaviors, policy, environment, and roadway conditions	To illustrate variables that influence drugged driving behaviors, road environment and traffic safety policies	Researchers reviewed the literature and published data is determine parameters and a stock-flow diagram was used to create the CLD and quantitative expressions were derived for simulation modeling	A component of system dynamics modeling to provide insight into the dynamic complexity of the drugged driving environment and traffic safety policy
Bensberg [35]	To describe the establishment of a multi-community chronic disease prevention initiative (Healthy Together Victoria) through a systems thinking lens	A way to address complex public health problems; holistic vs. reductionist perspective; the essence of a system is the causal connections between parts and feedback loops	To summarize findings and illustrate feedback loops	CLD was created by researchers from the analysis interview data	Identify strengths, limitations, and “possible remedies for the purpose of advancing health infrastructure initiatives and reforms.”
Bradley [34]	To report on the importance of employing systems thinking for the prevention and response to COVID-19	Society is a complex adaptive system with interconnected factors impacting the spread of infection; system structure influences system behaviour; systems change is needed to mitigate COVID-19	To visualize the causal connections and components of society to better understand feedback loops and relationships impacting the entire status of a system impacted by COVID-19	CLD was created by researchers only	Provide a visual example of the dynamic and complex interactions and systems changes needed to address COVID-19
Brereton [28]	To explore the complex causal relationships between children’s health, environment, social, and economic influences in least developed countries	A science that explores how parts connect, react, and interact to increase recognition of non-linearity and cause and effect relationships; to view the “forest and the trees.”	A tool to uncover root problems that are often difficult to view within complex systems; “Each CLD tells a story that links cause and effect through feedback” and that can be used to surface mental models and policy decisions among stakeholders	CLD was created from data on the most significant causes of childhood mortality and a narrative literature review	Highlight potential leverage points in children’s health and enable greater insight for policy and practice
Brown [48]	To present how a community used a CLD to track the underlying system changes resulting from implementing a healthy eating curriculum in a school	A method to address complexity	To present the relationships and variables that influence complex problems	CLD was created from seven group model building sessions where implementation strategies were tracked	Demonstrate how a CLD can be used to measure system changes and evaluate obesity prevention interventions
Burrell [36]	To develop a concept model of key causal structures driving dynamics of community violence escalation over time in a context of historical racism	to study complex problems as the manifestation of dynamic interactions among their constituent parts	To represent “dynamic hypotheses” about the system structure producing observed outcomes over time; illustrate complex interactions (e.g., interdependence, delays between cause and effect, mutual interaction, and feedback loops reinforcing or counteracting earlier changes)	CLDs were created from stakeholder interviews, documentaries, an ethnography, and a literature review	Convey new theoretical insights and implications regarding the interplay of factors for reducing violence escalation and disparity
Clarke [37]	To examine the dynamics and decisions regarding obesity prevention policy adoption within multi-community chronic disease prevention initiative (Healthy Together Victoria)	A non-linear and holistic perspective; appreciation of the multiple, interacting forces guiding policy decisions; understanding system behavior in terms of structures and patterns and feedback mechanisms	A heuristic tool to help document interconnections, virtuous/vicious feedback mechanisms, and leverage points to inform strategies for systems change	CLD was created by researchers using data from interviews, documents, and fields notes	Enhance theoretical analysis of obesity prevention policy and demonstrate feedback loops and leverage points that either spurred or resisted obesity prevention policy
Crielaard [26]	To model social norms regarding body weight and obesity prevalence using system dynamics modeling	A complex system is non-linear, is more than the aggregation of its parts, and has feedback loops that influence emergent system behaviour	To conceptualize the system and inform system dynamic modeling; a means to simulate “what if” scenarios and emergent system behavior to better understand the causal links and variables at play	CLD was created after conducting interviews with experts and the causal links found during the interviews were confirmed via literature review	Inform simulation scenarios and policy decision-making for group-level obesity interventions
Eker [27]	To combine quantitative simulation modelling, an interpretivist approach, and a participatory method to examine housing, energy and wellbeing aspects of the UK’s housing stock	To understand the dynamic behavior of complex systems or the systems underlying a policy problem and causal feedback thinking and non-linearity among elements; to examine the complexity of interactions between housing, energy and wellbeing	A tool in system dynamic modeling	CLD created as part of participatory system dynamics (SD) modelling that included stakeholder interviews and group model building	For simulation modelling and results were to inform policy debates
Gerritsen [41]	To describe group model building and system mapping methods used to study fruit and vegetable intake among children and evaluate effectiveness of various tools (graphs over time, cognitive mapping and CLDs)	A way to address complex problems characterized as having multiple causes, multilevel contexts, no single solution, and requiring multisectoral action	To increase understanding of the dynamics of complex problems and system behavior, the feedback mechanisms at play, and the determinants of fruit and vegetable intake among children	CLD created from data obtained through group model building	Identify system change actions and increase understanding about complex systems and systems thinking
Hassmiller Lich [46]	To report on group concept mapping and system dynamics modeling as complimentary methods to address complex problems in evaluation and strategic planning	A way to increase understanding of interconnected factors and cause and effect relationships that influence public health, social, behavioral, or environmental problems	To engage stakeholders in identifying and visualizing cause-effect relationships among variables	CLD created from data collected through group concept mapping	Identify leverage points for strategic planning and intervention scenarios
Jalali [38]	To increase understanding of the effectiveness of obesity prevention interventions from an endogenous, organizational behavior or dynamics perspective and use system dynamic modeling methods	To study the complexity or dynamics of program success and failure	A step in system dynamic modeling	CLD created from interview data and published data	Help monitor and improve the design and implementation of interventions in order to avoid the dynamics that lead to poor outcomes
Klement [29]	To include individual level factors that influence COVID-19 to Sahin et al’s (2020) CLD of environmental-health-socio-economic systems of the COVID-19 pandemic	To increase understanding the interconnections among parts of a system, feedback loops, system structure and behavior at multiple levels	A step in system dynamic modeling	CLD created from researcher knowledge, evidence, and assumptions	Illustrate the complexity of COVID-19
Knai [30]	To demonstrate the application of a complex systems approach to analyze the commercial determinants of health in terms of problem identification and policy development	Attends to heterogenous stakeholders and interventions, their dynamic interactions at multiple levels, adaptation and emergent system behavior, nonlinearity, feedback loops, and power dynamics in systems in order to influence systems to be more health promoting	To illustrate the complexity of COVID-19 and the effectiveness of public health measures	CLD created from researcher knowledge, evidence and assumptions	Identify interventions and further research that highlights the interdependence among variables such as market and nonmarket strategies and sectors and how they work together to form system behavior with respect to commercial determinants of noncommunicable diseases
Maitland [42]	To report on study protocol for applying a ‘whole of system approach’ to evaluate strategies to address childhood obesity	To examine complexity, nonlinearity, relationships among variables, and feedback loops	To visualize the system by illustrating system components and interconnections that results in a narrative about a problem	Study protocol; Did not create a CLD	Study protocol; Did not create or use a CLD.
Osman [31]	To report on applying systems thinking methods and tools to identify interdependence and underlying factors that influence TB.	Health systems are complex adaptive systems; many interactions among parts produces system behaviour	One component of a larger study to focus on nonlinearity of relationships among factors, feedback loops, and changes in context	CLD created from a seminar with diverse experts; utilized fishbone analysis, a 5 whys approach, and affinity diagrams	To develop implementation action plans, risk mitigation strategies and track changes in the system
Owen [39]	To report on applying systems thinking and feedback loops to create a CLD to visualize and understand the dynamic complexity of a successful intervention to address childhood obesity	To understand system structure, feedback loops, non-linearity, delays, system behavior, factors that influence complex problems, and to identify interventions	To identify and share understanding of system elements and nonlinear system structures that influences or dictates system behaviour	CLD created from interview data	To evaluate project implementation in order to understand leverage points to strengthen systems and/or create new systems
Parmar [40]	To study roles of community health volunteers in managing diabetes and hypertension among Syrian refugees and recommend improvements	To examine complex adaptive systems in terms of non-linear interactions among multiple actors and processes	To understand complex systems and impact and/or consequences of changes to programming	CLD created from document review, key informant interviews, and workshops	To identify issues and strategies for improving the community health workers program
Riley [43]	To report on a novel combination of systems methods and tools and systemic inquiry processes in a study of community-based chronic disease prevention	An analytic or conceptual lens (to study three organizing principles: interdependent relationships, perspectives and boundaries); systemic inquiry as a process to build capacity for ongoing action learning	To support community members and researchers to examine influencing factors of local systemic problems with respect to chronic disease prevention	CLD created from group modeling building in each participating community	To highlight feedback loops that are either reinforcing or balancing and identify places to intervene
Sahin [32]	To visualize the complexity in managing the COVID-19 pandemic through a systems lens by identifying the interconnectivity between health, economic, social and environmental aspects	A framework to better understand the big picture through identifying the multi-faceted consequences of decisions and to design the most effective strategies to manage the impacts of unintended consequences	To identify and illustrate feedback relationships and pinpointed leverage points	CLD created from researchers existing knowledge, geographical data, and government documents via four expert workshops	To identify leverage points to address COVID-19
Swierad [44]	To describe how group model building was conducted and report on the findings with respect to childhood obesity	Obesity is discussed in terms of a complex, multi-level problem	To support community members to understand concepts and tools of system dynamics and systems thinking	CLD created from group model building	To illustrate and increase understanding of childhood obesity as a multifactorial problem (e.g., sociocultural factors), tailor culturally sensitive interventions, and generate hypotheses for further research
Urwannachotima [33]	To study the dynamic interactions among variables associated with sugar-sweetened beverage tax and dental caries in Thailand	To take a whole system perspective of dynamic interactions	To visualize dynamic interactions or relationships among variables and their interdependence	CLD created from in-depth interviews and group model building	The potential of the CLD lies with quantitative modeling and formulating recommendations for intervention

### Research Aims

Although the literature addressed a range of public health topics, non-communicable disease prevention was most frequently addressed (15/23) and of those, seven were focused on obesity prevention. Table 2 provides a list of research topics.

**TABLE 2:** Research topics of reviewed literature (Canada, 2021).

Research topic	Citation
Children’s Health	[28]
Community Violence	[36]
COVID-19	[29, 32, 34]
Driving Behavior	[45]
Evaluation	[46]
Housing	[27]
Noncommunicable disease prevention	
Commercial Determinants of Health	[30]
Diabetes/Hypertension/Community Health Workers	[40]
Healthy Schools	[48]
Prevention Systems	[35, 43]
Sugar Sweetened Beverage Tax	[33]
Obesity Prevention: Organizational dynamics	[38]
Obesity Prevention: Weight-Related Behavior	[26]
Obesity Prevention: Children	[39, 44, 47]
Obesity Prevention: Fruit and Vegetable Intake	[41]
Obesity Prevention: Policy	[37]
Obesity Prevention: Whole of system	[42]
Tuberculosis	[31]

In terms of research aims found in the 23 articles, four themes emerged: 1) to examine the complexity of a public health topic and illustrate complex systems thinking [26–34]; 2) to discuss the complexity of a public health intervention [35–40]; 3) to describe study protocol and how CLDs were created [41–44]; and 4) to illustrate how CLDs can be used to monitor and track initiatives to improve population health or evaluate impact of interventions [45–48].

### Complex Systems Thinking

Complex systems thinking was discussed in terms of systems, problems, interventions, and key concepts that drive this type of approach. Several articles indicated that the *systems* they were studying were complex, for example:

A complex system may be characterized by its heterogeneity (various actors and structures at different levels); its dynamic, interactive, and adaptive nature (its ability to respond to or resist external changes, or changes in the interacting parts); and its emergent properties (arising through interactions between processes or factors that alone do not exhibit such properties) [30].

Following on this, *feedback loops in complex systems* were explicitly discussed in all articles to some extent. Jalali et al. [38] described these in terms of “causal chains of multiple variables in which changes in each variable could be traced back to its historical values.” They go on to define the difference between reinforcing and balancing feedback loops.

Another way complex systems thinking was described was with respect to complex *problems* and *interventions*. Burrell et al. [36] discussed community violence in terms of embedded contexts and the lack of holistic understanding of such “dynamic complexity.” Complex problems and interventions were often discussed together. The need to move away from “isolated intervention thinking” to systemic interventions to study systems change was highlighted by Knai et al. [30].

All articles built upon the descriptions reported above in some manner when discussing *complex systems thinking.* Some articles described this as providing “the opportunity to understand, test, and revise our understanding of how the different components in a system work together” [31] and “to study complex problems as the manifestation of dynamic interactions among their constituent parts” [36]. Furthermore, a few articles expanded the discussion to include such concepts as boundary judgement [38, 43, 47], that is, “establishing boundaries to the system is a fundamental starting point to efforts to change systems” [47].

### Why Causal Loop Diagrams?

CLDs were mostly seen as a means or a tool to examine feedback at play in public health issues. Some articles were explicit [28, 32, 33, 40, 43, 44] while others implied this. Both Riley et al. [43] and Parmar et al. [40] labeled this as “causal loop analysis” and the resulting CLDs were a means to understand systems and potential “programming.” Using a CLDs was a new tool for some [42, 46] and as one article related, “business as usual” was not working to address obesity [47]. CLDs were also considered a tool to help tell a story. For example, a CLD was thought to support the development of “a concise narrative about a particular problem” [42] and Brereton et al. [28] stated that “every causal loop tells a story that links cause and effect through feedback.”

### How Were Causal Loop Diagrams Created?

There were many combinations of methods used to create CLDs. In this section we present this diversity in terms of 1) data sources, 2) processes, 3) data analysis, 4) frameworks, and 5) diagramming (Table 3).

**TABLE 3:** How causal loop diagrams were created (Canada, 2021).

First Author/Citation	Data used for CLD creation			Process used for CLD creation		
	Primary data	Secondary data	Researcher knowledge	Researcher created only	Researcher created with stakeholder refinement	GMB with Stakeholders
Allender [47]	✓					✓
Araz [45]		✓		✓		
Bensberg [35]	✓			✓		
Bradley [34]			✓	✓		
Brereton [28]		✓		✓		
Brown [48]	✓					✓
Burrell [36]	✓	✓		✓		
Clarke [37]	✓	✓		✓		
Crielaard [26]	✓	✓		✓		
Eker [27]	✓					✓
Gerritsen [41]	✓					✓
Hassmiller Lich [46]	✓					✓
Jalali [38]	✓	✓		✓		
Klement [29]		✓		✓		
Knai [30]		✓		✓		
Maitland [42]	✓					✓
Osman [31]	✓					✓
Owen [39]	✓				✓	
Parmar [40]	✓	✓			✓	
Riley [43]	✓					✓
Sahin [32]	✓	✓				✓
Swierad [44]	✓					✓
Urwannachotima [33]	✓					✓

#### Data Sources

Both primary and secondary data were used for creating CLDs (Table 3). Most articles reported on primary data collection (18/23) and this included interviews [26, 27, 33, 35–40], group model building with stakeholders and/or community members [32, 41, 43, 44, 46, 48], behavioral data [42, 47], fieldnotes [37], and workshops with experts [31]. Twelve articles used primary data only.

Secondary data was used in 10 articles [26, 28–30, 32, 36–38, 40, 45] and this consisted of document and/or literature review (Table 3). Of the eighteen articles that reported on primary data collection, six included document review [26, 32, 36–38, 40]. Documents included policy briefings, reports, consultation papers, and evaluation reports [37], documentaries and ethnographies [36], program data [38], geographical information and government documents [32], and data from published databases [28, 37, 45]. Literature reviews were undertaken in four articles and these either supplemented primary data [26], secondary data [28, 45], or both [36]. Document and literature review were utilized in four articles [28–30, 45].

#### Processes

There were three processes used to create CLDs: group model building, researcher created only, and researcher created with stakeholder refinement (Table 3). Group model building (GMB) was the most common process as reported in 11 articles [27, 31–33, 41–44, 46–48]. Urwannachotima et al. [33] described GMB as “an established methodology for engaging stakeholders to gain mutual understanding of complex relationships and to collectively develop comprehensive systems models that represent the cause and effect relationships of a problem.” They go further to explain that “stakeholders are deeply and actively involved in the process of model construction through the exchange, assimilation, and integration of mental models into a holistic system description.” GMB was generally reported to be a process where participants brainstormed and named potential variables, drew connections and feedback loops between the identified variables, and then mapped these ideas onto a final CLD. However, there was a variety of GMB processes used and was often not clearly described in terms of session design and activities. Beyond GMB, Hassmiller Lich et al. [46] discussed group concept mapping and Gerritsen et al. [41] described graphing over time and cognitive mapping.

CLDs created by researchers only was the second most common process (10/23). Two articles reported that CLDs were presented to stakeholders for refinement [39, 40]. The range of approaches included:

• Using coded interview data to map interactions between key variables [26, 35–38],
• Conducting a literature review to compare causal links uncovered in interview data [26] or a document review [29, 30],
• Completing both a literature review and a document review to identify variables [28, 45],
• Building on an existing CLD [29], and
• Creating a CLD solely from researcher knowledge and expertise [34].

#### Data Analysis

Overall, we found that description was often lacking regarding qualitative data analysis methods used. However, some articles [35, 37, 39] that collected primary data discussed methods described by Kim and Anderson [49]. Others such as Owen et al. [39] created a table to demonstrate how they used coded interview transcript statements to inform their CLD. Steps in the analysis included 1) using coded text to show causal linkages, 2) translating these to cause-and-effect variables, and 3) creating word-and-arrow diagrams for CLD use. Similarly, Brereton and Jagals [28] presented a table to identify variables and describe influencing links.

#### Frameworks

Several articles applied specific frameworks to inform research. For example, Allender et al. [47] used Foster-Fishman’s [50] theoretical framework of six elements (i.e., systems norms, financial resources, human resources, social resources, regulations, and operations) to study root causes, system interactions, and levers for change. Similarly, Baugh Littlejohns and Wilson’s [5] framework of seven attributes of effective prevention systems (i.e., leadership, resources, health equity paradigm, information, implementation of desired actions, complex systems thinking, collaborative capacity) was used by Bensberg et al. [35] in their study design.

#### Diagramming

Many articles reported on the use of software for creating the actual diagram. Vensim [31, 35, 37, 39, 40, 44–46], Stella Architect [28], and STICK-E [43] were the three diagrammatic programs used. Further to the actual diagram, there was a wide array of CLD types and degrees of diagram readability. We found that some CLDs were kept quite simple, with fewer variables, arrows, and loops, while others were very complicated. For example, Brereton et al. [28] created a tightly packed and dense color-coded main CLD and six diagrams of various feedback loops to highlight key variables, relationships, and potential leverage points. Overall, we found that key variables in blocks or shapes, labelled arrows and feedback loops, color coding, legends, and clear diagram interpretation descriptions were important aspects for readability.

### Intended Uses of Causal Loop Diagrams

There were nine ways that CLDs were intended to be used and these are identified in Table 4. The following provides examples of each intended use.

**TABLE 4:** How causal loop diagrams were intended to be used (Canada, 2021).

First author/citation	How were CLDS primarily intended to be used								
	Inform policy	Identify leverage points systems change	Inform practice	For system dynamic modelling	Measure or evaluate	Stakeholder engagement to take action	To illustrate complexity	To inform future research	To enhance theory
Allender [47]			✓			✓			
Araz [45]	✓			✓					
Bensberg [35]	✓	✓	✓						
Bradley [34]	✓	✓							
Brereton [28]	✓	✓	✓						
Brown [48]					✓				
Burrell [36]	✓	✓	✓						✓
Clarke [37]	✓	✓	✓						✓
Crielaard [26]	✓		✓	✓					
Eker [27]	✓			✓			✓		
Gerritsen [41]		✓					✓		
Hassmiller Lich [46]		✓				✓			
Jalali [38]	✓		✓	✓					
Klement [29]							✓		
Knai [30]		✓	✓				✓	✓	
Maitland [42]					✓		✓	✓	
Osman [31]		✓	✓		✓				
Owen [39]			✓		✓				
Parmar [40]	✓	✓	✓						
Riley [43]		✓	✓						
Sahin [32]		✓					✓		
Swierad [44]	✓		✓			✓		✓	
Urwannachotima [33]	✓			✓			✓		
Allender [47]			✓			✓			
Araz [45]	✓			✓					·
Bensberg [35]	✓	✓	✓						
Bradley [34]	✓	✓							
Brereton [28]	✓	✓	✓						
Brown [48]					✓				
Burrell [36]	✓	✓	✓						✓
Clarke [37]	✓	✓	✓						✓
Crielaard [26]	✓		✓	✓					
Eker [27]	✓			✓			✓		
Gerritsen [41]		✓					✓		
Hassmiller Lich [46]		✓				✓			
Jalali [38]	✓		✓	✓					
Klement [29]							✓		
Knai [30]		✓	✓				✓	✓	
Maitland [42]					✓		✓	✓	
Osman [31]		✓	✓		✓				
Owen [39]			✓		✓				
Parmar [40]	✓	✓	✓						
Riley [43]		✓	✓						
Sahin [32]		✓					✓		
Swierad [44]	✓		✓			✓		✓	
Urwannachotima [33]	✓			✓			✓		

#### Illustrate Complexity and Identify Leverage Points

Illustrating complexity was aligned with research aims in several articles (Table 4) and was implicit in the other articles with respect to using CLDs. Identifying leverage points was explicitly discussed in twelve articles. Osman et al. [31] found that key variables and their interactions pointed to strategies to enhance leadership “through a reduction in bureaucracy in the health system.” Similarly, Bensberg et al. [35] identified leadership as a leverage point as well as knowledge and data, resources, workforce, and collaborative relationships that need to be “nudged in the desired direction.” One of the more detailed descriptions of leverage points was from Sahin et al. [32]. They adapted Meadows [51] framework of places to intervene in system to identify shallow or deep leverage points to address the “wicked complexity” of the COVID-19 pandemic.

#### Inform Policy and Practice

Informing policy was a reported intended use of CLDs in twelve articles (Table 4). Some articles were detailed in offering policy directions while others simply stated that the CLD could inform policy. Clarke et al. [37] examined “key influences on policy processes, and to identify potential opportunities to increase the adoption of recommended policies” with respect to a state government obesity prevention initiative. Other examples include the need for policies to address population growth, family size, and family planning to improve child health [28], housing, energy and wellbeing [27], and sugar-sweetened beverage tax to reduce sugar consumption and dental caries [33].

Informing practice was also a frequently identified intended use of CLDs (13/23) (Table 4). For example, Osman et al. [31] stated that their CLD could be used “to develop local action plans for implementation and consider strategies for mitigating possible future risks” and Parmar et al. [40] to develop “strategies to enhance capacities, services, and coordination to improve the health of refugees.”

#### For System Dynamics Modeling

Five articles created CLDs for use in system dynamics modeling [26, 27, 38, 45] (Table 3). This was defined by Araz et al as “a computer-aided approach to model and facilitate analysis of complex system behaviors over time” [45]. They further described the steps in system dynamic modeling, and this was very much in line with other articles:

We first constructed a causal loop diagram (CLD) informed by the existing literature to present the causal relationships between variables in drugged driving behaviors and traffic safety policies. A stock-flow diagram (SFD) was then used to convert these dynamic processes into quantitative expressions and a simulation tool [45].

Mirroring the above descriptions, Crielaard et al. [26] discussed the value of system dynamic modeling in terms of testing policy options from “studying ‘what if’ scenarios using computational modelling approaches.” It was notable that Urwannachotima et al. [33] and Swierad et al. [44] stated that the primary value of CLDs was in quantitative modelling.

#### Measure and Evaluate Systems Change

Table 4 identifies four articles that used CLDs to help measure and evaluate systems change [31, 39, 42, 48]. For example, Owen et al. [39] reported that “the methods provide a technique to retrospectively evaluate community interventions from a systems perspective and understand the way successful and unsuccessful interventions addressed complexity.” They go further to explain that CLDs go beyond linear cause and effect logic models used in traditional evaluation and lessons regarding unintended consequences provide insights “to increase the chances of success for new prevention initiatives.”

#### Enhance Stakeholder and Community Participation

As discussed above, group model building (GMB) was a frequently reported process to create CLDs and inherent in these processes was the desire for stakeholder and/or community participation and shared understanding (Table 4). Gerritsen et al. [41] stated what many others did, that is, GMB helped people develop an understanding of the system under study and that “participants learn to see causal connections and how these connections result in patterns of behaviour evolving over time.” They hypothesized that resulting plans for system change would be more successful with this fundamental level of participation and understanding. Another article highlighted that GMB brought diverse stakeholders “together to develop a system understanding of the problem, thus paving the way for further collaboration and community action” [44].

#### Inform Future Research and Enhance Theoretical Perspectives

The final two intended uses of CLDs were to inform future research and enhance theoretical perspectives (Table 4). These intended uses were not widely discussed and if at all, they were mostly short aspirational statements. However, one example where future research was explicitly discussed was provided by Swierad et al. [44]. Here they reported that “hypotheses” from a CLD of childhood obesity could be used in future research such as “impact of food eaten at school influencing norms and acceptability of western/packaged food, elasticity of grandparents’ food norms, diversity of grandparents’ ideal body image for children, or beliefs in health of traditional foods.”

With respect to using CLDs to enhance theoretical perspectives, Clarke et al. [37] suggested that the CLD “enhanced previously published theoretical analyses of obesity prevention policy decision-making systems by making explicit how underlying feedback loops either spurred policy change or resistance.” Another example is from Burrell et al. [36]. They reported that creating a CLD resulted in “a testable ecologically oriented theory of violence” and “the resulting model conveys new theoretical insights on how racial and economic features of urban settings interact with intrapsychic dimensions to create a self-perpetuating system of violence.”

## Discussion

This section answers our second research question: What recommendations emerge regarding how to create and use CLDs in public health research? We offer nine learnings from the results above and interweave ideas from other research to support preliminary recommendations or possible directions to take forward in future research.

### Boundary Judgements

We learned that some articles described in detail theoretical orientations with respect to complex systems thinking while others gave brief explanations. The most frequent concepts regarding complex systems were the inherent dynamic interactions among many entities, factors, variables that illustrate whole system structure and behavior. This is consistent with other public health literature on the topic [52–54]. The difference in descriptions was more a matter of comprehensiveness than definitions. For example, boundary judgement was not well articulated in the articles. According to Ulrich [55], drawing boundaries builds in selectivity and partiality and therefore transparency is important in study design. Therefore, we recommend that attention be given to defining boundaries to signal a specific endogenous perspective and a unique, snap-shot-in-time diagram of feedback loops of system behavior [56].

### From Theory to Leverage Points

Some articles had strong theoretical coherence with respect to complex systems thinking that was demonstrated in discussions about the reasons for choosing, creating, and using CLDs. We learned that articles were most coherent when they first discussed feedback loops from a theoretical perspective and then carried this through to creating CLDs and to using them to identify leverage points for systems change (see for example 30). Overall, the descriptions of feedback in the articles were aligned with the idea that CLDs are “the applications of the loop concept underlying feedback and mutual causality” and that feedback loops are “powerful unifying notions that illuminate the structure of arguments, explanations, and causal views” [56]. Meadows [51] is well-known for explaining that disrupting or amplifying feedback loops can be effective leverage points in systems change. Therefore, we recommend that future research be designed with this theoretical coherence in mind.

### Theoretical Frameworks

Lewin’s famous statement that “there is nothing so practical as good theory” was salient for what we learned [57]. Few articles used theoretical frameworks in research design or discussed the need to advance theory (i.e., complexity, systems) in public health research. The articles that used frameworks appeared to be more robust especially with respect to embedding theoretical constructs in the resultant CLD (see for example 35). While we appreciate that theory is emerging, we recommend that this be given more emphasis to help continue to build a solid foundation for furthering the application of CLDs in public health research.

### Qualitative Data Analysis

Knai et al. [30] pointed out that current public health research “concentrates mainly on a system’s elements rather than the interconnections within it, and this is beginning to reveal its intrinsic limitations.” Some articles described data analysis methods to identify variables and examine interconnections to draw CLDs, however, others lacked clear descriptions of the often highly iterative methods and therefore it was difficult to follow a data trail and assess the resultant CLD. We recommend that more clarity be provided as to how researchers innovate in qualitative data analysis to further develop the art and science of creating CLDs.

### Mixed Methods

We found a range of research methods used to create CLDs. Ozawa et al. [58] state that mixed methods research is important

because it allows researchers to view problems from multiple perspectives, contextualize information, develop a more complete understanding of a problem, triangulate results, quantify hard-to-measure constructs, provide illustrations of context for trends, examine processes/experiences along with outcomes and capture a macro picture of a system.

We hypothesize that mixed methods may produce more robust CLDs, however, this needs to be examined. We recommend that future research be undertaken to assess the strengths, limitations, and benefits of using mixed methods and determine what methods create greater confidence in the variables and feedback loops illustrated in CLDs.

### Participatory Action Research

We found there was a wide range of *who* was involved in creating CLDs, from researchers only to multiple group model building sessions with stakeholders and community members. We see the latter methodology embedded in the traditions of action research [59] and/or community-based participatory research (CBPR) [60]. The CBPR approach involves “a commitment to conducting research that shares power with and engages community partners in the research process” and is intended “to increase knowledge and understanding of a given phenomena and integrate knowledge gained with interventions and policy and social change” [60]. There was little discussion of CBPR in the articles. We recommend that greater engagement with participatory action research literature be undertaken to embed the theory and philosophy of genuine participation and empowerment in research and action.

### Knowledge Translation

There was limited discussion regarding how exactly CLDs were to be used to enhance evidence-informed policy and practice. Few articles explicitly discussed incorporating knowledge users or those able to use research results. As Sturmberg [61] relates, this requires users who are “deeply interested in understanding the highly interconnected and interdependent nature of the issues.” This led us to think about the importance of knowledge translation (KT) and how to strengthen the use of CLDs. Haynes et al. [6] state that KT needs to be conceptualized as not “a discrete piece of work within wider efforts to strengthen public health, but as integral to and in continual dialogue with those efforts.” We recommend that future public health research using CLDs should articulate KT plans that articulates knowledge user engagement in defining outcomes for strengthening public health policies and practices.

### Health Equity

We conceptualize public health research to be guided by principles of social justice and human rights to address the goal of reducing health inequities through action on the determinants of health. Although many articles discussed determinants of health, the goal of reducing health inequities was largely absent. Baum et al. [62] discuss the concept of path dependency as “the tendency of institutions to retain policy directions and preferences rather than change or reform them.” They further suggest that disrupting “path dependency that exacerbates health inequities” is critical and we see how CLDs could uncover path dependencies. We recommend that CLDs in public health research should include the examination of leverage points for pro-equity policy and practice.

### The Diagram

Senge [63] states that “reality is made up of circles” but often arguments and explanations are linear, therefore, CLDs can provide “a language of interrelationships” to uncover deep patterns in systems. Studying the interrelationships and explanations of each CLD was outside the scope of this paper, however, we learned about some basic elements of reader friendly CLDs. We recommend that the following questions could be used assess CLDs: Are established conventions [56] used effectively for drawing the CLD (e.g., labeling, positive and negative arrows, reinforcing and balancing loops)? Does the diagram illuminate the most significant variables, feedback loops or leverage points? How well does the diagram function as an effective medium for presenting findings to knowledge users? How well does the CLD tell a story of what’s going on in a system?

### Strengths and Limitations

In terms of limitations, the 23 articles were not considered to be comprehensive. Since completing the study, we found that Mui and others [64] published an article on a community-based system dynamics approach and suggests solutions for improving healthy food access in a low-income urban environment. We also found Savona et al. [65] identified the views of adolescents regarding the causes of obesity and used CLDs. While this can be considered a limitation, we hope to see a continual building of knowledge and skill in using CLDs in public health research. A strength of this paper is that 23 recent articles were identified that used CLDs and the depth and breadth of discussion in the articles provided good representation. Having three authors conduct the literature review is also a strength because this afforded a high degree of confidence in reporting results and transparency in search strategy and data extraction, analysis and synthesis. Together the results and recommendations can contribute to informing global public health research by highlighting key considerations to help design research and address public health issues through complex systems thinking.
